# ﻿*Sinocyclocheilusxiejiahuai* (Cypriniformes, Cyprinidae), a new cave fish with extremely small population size from western Guizhou, China

**DOI:** 10.3897/zookeys.1214.127629

**Published:** 2024-10-03

**Authors:** Cui Fan, Man Wang, Jia-Jia Wang, Tao Luo, Jia-Jun Zhou, Ning Xiao, Jiang Zhou

**Affiliations:** 1 School of Karst Science, Guizhou Normal University, Guiyang 550001, Guizhou, China; 2 Institute of Hydrobiology, Chinese Academy of Sciences, Wuhan 430072, Hubei, China; 3 University of Chinese Academy of Sciences, Beijing 100049, China; 4 School of Life Sciences, Guizhou Normal University, Guiyang 550025, Guizhou, China; 5 Zhejiang Forest Resource Monitoring Center, Hangzhou 310020, Zhejiang, China; 6 Guiyang Healthcare Vocational University, Guiyang 550081, Guizhou, China

**Keywords:** Cavefish, new species, morphology, phylogeny, taxonomy

## Abstract

This study describes a new species, *Sinocyclocheilusxiejiahuai***sp. nov**., discovered within a cave located in Hongguo Town, Panzhou City, Guizhou Province, southwestern China, with the type locality in the Nanpanjiang River basin. Phylogenetic trees reconstructed based on mitochondrial genes show that the new species represents an independent evolutionary lineage with large genetic differences, 1.9%–13.8% in mitochondrial Cyt *b*, from congeners. Morphologically, this species can be differentiated from the 79 species currently classified under the genus *Sinocyclocheilus* by several characteristics: absence of horn-like structures and indistinct elevation at the head-dorsal junction, absence of irregular black markings on the body lateral and scaleless, eyes large, eye diameter 13% of head length, dorsal-fin rays, iii, 6½, last unbranched ray strong, with serrations along posterior margin, pectoral-fin rays, i, 13, anal-fin rays, iii, 5, pelvic-fin rays, i, 7, lateral line pores 74, gill rakers well developed, nine on first gill arch, pectoral fins short, tip not reaching to pelvic-fin origin. The number of *Sinocyclocheilus* species has been increased from 79 to 80 since the description of this new species.

## ﻿Introduction

**Figure 1.**
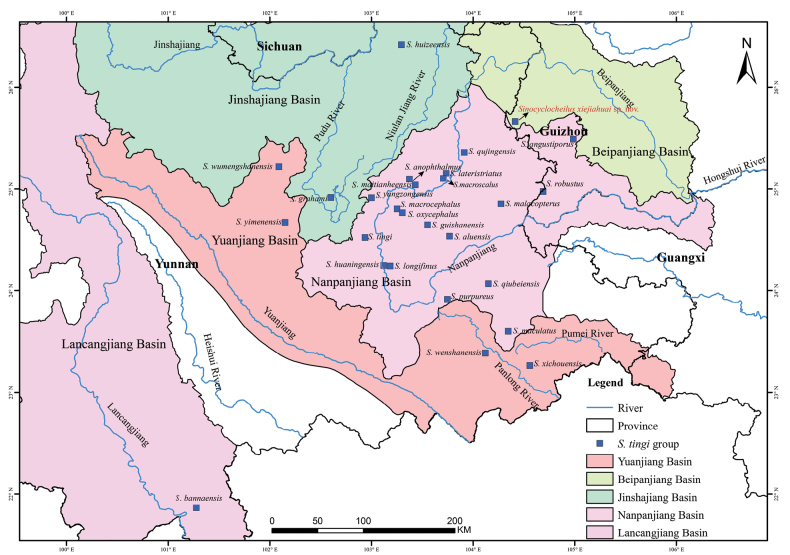
Sampling collection localities and distribution of the *Sinocyclocheilusxiejiahuai* sp. nov. and 26 species of the *S.tingi* species group of the genus *Sinocyclocheilus* in southwest China. The base maps are from Standard Map Service website (http://211.159.153.75/).

The genus *Sinocyclocheilus* Fang, 1936 (Cypriniformes: Cyprinidae) is endemic to China, and is currently found only in the Yangtze, Pearl, Lancangjiang, and Yuanjiang rivers (Xu et al. 2023). Based on recent taxonomic and phylogenetic studies, the genus *Sinocyclocheilus* includes 79 valid species (Shan et al. 2000; Zhao and Zhang 2009; Zhang et al. 2016; Luo et al. 2023; Xu et al. 2023; Shao et al. 2024), most of which are classified into five species groups, i.e., *S.angularis* species group includes 21 valid species, *S.cyphotergous* species group includes 20 valid species, *S.microphthalmus* species group includes three valid species, *S.jii* species group includes five valid species, and *S.tingi* species group includes 26 valid species (Zhao and Zhang 2009; Wen et al. 2022; Luo et al. 2023; Xu et al. 2023; Shao et al. 2024) (Table 1). The *S.angularis*, *S.cyphotergous*, and *S.microphthalmus* species groups have stygobite morphology, and the *S.jii* and *S.tingi* species groups a mixture of stygobite and stygophile morphology (Zhao and Zhang 2009). There are 25 species currently recorded for the *S.tingi* species group, of which 18, four, two, and one are distributed in the Nanpanjiang, Yuanjiang, Jinshajiang, and Lancangjiang rivers, respectively, and the new species is distributed in the Beipanjiang River (Fig. 1) (Zhao and Zhang 2009; Luo et al. 2023; Xu et al. 2023).

**Table 1.** List of 79 currently recognized species of the genus *Sinocyclocheilus* endemic to China and references. Recognized species modified from Jiang et al. (2019) and Xu et al. (2023).

ID	Species	Species group	Province	Rivers	Literature obtained
1	*S.altishoulderus* (Li & Lan, 1992)	*S.angularis* group	Guangxi	Hongshui River	Li and Lan 1992
2	*S.anatirostris* Lin & Luo, 1986	*S.angularis* group	Guangxi	Hongshui River	Lin and Luo 1986
3	*S.angularis* Zheng & Wang, 1990	*S.angularis* group	Guizhou	Beipanjiang River	Zheng and Wang 1990
4	*S.aquihornes* Li & Yang, 2007	*S.angularis* group	Yunnan	Nanpanjiang River	Li et al. 2007
5	*S.bicornutus* Wang & Liao, 1997	*S.angularis* group	Guizhou	Beipanjiang River	Wang and Liao 1997
6	*S.brevibarbatus* Zhao, Lan & Zhang, 2009	*S.angularis* group	Guangxi	Hongshui River	Zhao et al. 2009
7	*S.broadihornes* Li & Mao, 2007	*S.angularis* group	Yunnan	Nanpanjiang River	Li and Mao 2007
8	*S.convexiforeheadus* Li, Yang & Li, 2017	*S.angularis* group	Yunnan	Nanpanjiang River	Yang et al. 2017
9	*S.hyalinus* Chen & Yang, 1994	*S.angularis* group	Yunnan	Nanpanjiang River	Chen et al. 1994
10	*S.longicornus* Luo, Xu, Wu, Zhou & Zhou, 2023	*S.angularis* group	Guizhou	Nanpanjiang River	Xu et al. 2023
11	*S.jiuxuensis* Li & Ran, 2003	*S.angularis* group	Guangxi	Hongshui River	Li et al. 2003c
12	*S.flexuosdorsalis* Zhu & Zhu, 2012	*S.angularis* group	Guangxi	Hongshui River	Zhu and Zhu 2012
13	*S.furcodorsalis* Chen, Yang & Lan, 1997	*S.angularis* group	Guangxi	Hongshui River	Chen et al. 1997
14	*S.mashanensis* Wu, Liao & Li, 2010	*S.angularis* group	Guangxi	Hongshui River	Wu et al. 2010
15	*S.rhinocerous* Li & Tao, 1994	*S.angularis* group	Yunnan	Nanpanjiang River	Li and Tao 1994
16	*S.simengensis* Li, Wu, Li & Lan, 2018	*S.angularis* group	Guangxi	Hongshui River	Wu et al. 2018
17	*S.tianeensis* Li, Xiao & Luo, 2003	*S.angularis* group	Guangxi	Hongshui River	Li et al. 2003d
18	*S.tianlinensis* Zhou, Zhang, He & Zhou, 2004	*S.angularis* group	Guangxi	Nanpanjiang River	Zhou et al. 2004
19	*S.tileihornes* Mao, Lu & Li, 2003	*S.angularis* group	Yunnan	Nanpanjiang River	Mao et al. 2003
20	*S.xingyiensis* Luo, Tang, Deng, Duan & Zhang, 2023	*S.angularis* group	Guizhou	Nanpanjiang River	Luo et al. 2023
21	*S.zhenfengensis* Liu, Deng, Ma, Xiao & Zhou, 2018	*S.angularis* group	Guizhou	Beipanjiang River	Liu et al. 2018
22	*S.anshuiensis* Gan, Wu, Wei & Yang, 2013	*S.microphthalmus* group	Guangxi	Hongshui River	Gan et al. 2013
23	*S.longshanensis* Li & Wu, 2018	*S.microphthalmus* group	Yunnan	Nanpanjiang River	Li et al. 2018
24	*S.microphthalmus* Li, 1989	*S.microphthalmus* group	Guangxi	Hongshui River	Li 1989
25	*S.aluensis* Li & Xiao, 2005	*S.tingi* group	Yunnan	Nanpanjiang River	Li et al. 2005; Zhao and Zhang 2013
26	*S.angustiporus* Zheng & Xie, 1985	*S.tingi* group	Guizhou; Yunnan	Nanpanjiang River	Zheng and Xie 1985; Zhao and Zhang 2009
27	*S.anophthalmus* Chen & Chu, 1988	*S.tingi* group	Yunnan	Nanpanjiang River	Chen et al. 1988a
28	*S.bannaensis* Li, Li & Chen, 2019	*S.tingi* group	Yunnan	Lancangjiang River	Li et al. 2019
29	*S.grahami* (Regan, 1904)	*S.tingi* group	Yunnan	Jinshajiang River	Zhao and Zhang 2009
30	*S.guishanensis* Li, 2003	*S.tingi* group	Yunnan	Nanpanjiang River	Li et al. 2003a
31	*S.huaningensis* Li, 1998	*S.tingi* group	Yunnan	Nanpanjiang River	Li et al. 1998
32	*S.huizeensis* Cheng, Pan, Chen, Li, Ma & Yang, 2015	*S.tingi* group	Yunnan	Jinshajiang River	Cheng et al. 2015
33	*S.lateristriatus* Li,1992	*S.tingi* group	Yunnan	Nanpanjiang River	Li 1992
34	*S.longifinus* Li, 1998	*S.tingi* group	Yunnan	Nanpanjiang River	Li et al. 1998
35	*S.macrocephalus* Li,1985	*S.tingi* group	Yunnan	Nanpanjiang River	Li 1985
36	*S.macroscalus* Li, 1992	*S.tingi* group	Yunnan	Nanpanjiang River	Li 1992
37	*S.maculatus* Li, 2000	*S.tingi* group	Yunnan	Nanpanjiang River	Zhao and Zhang 2009; Li et al. 2000a
38	*S.maitianheensis* Li,1992	*S.tingi* group	Yunnan	Nanpanjiang River	Li 1992
39	*S.malacopterus* Chu & Cui, 1985	*S.tingi* group	Yunnan	Nanpanjiang River	Chu and Cui 1985
40	*S.oxycephalus* Li, 1985	*S.tingi* group	Yunnan	Nanpanjiang River	Li 1985
41	*S.purpureus* Li, 1985	*S.tingi* group	Yunnan	Nanpanjiang River	Li 1985
42	*S.qiubeiensis* Li, 2002	*S.tingi* group	Yunnan	Nanpanjiang River	Li et al. 2002b
43	*S.qujingensis* Li, Mao & Lu, 2002	*S.tingi* group	Yunnan	Nanpanjiang River	Li et al. 2002c
44	*S.robustus* Chen & Zhao, 1988	*S.tingi* group	Guizhou	Nanpanjiang River	Chen et al. 1988b
45	*S.tingi* Fang, 1936	*S.tingi* group	Yunnan	Nanpanjiang River	Zhao and Zhang 2009
46	*S.wenshanensis* Li, Yang, Li & Chen, 2018	*S.tingi* group	Yunnan	Yuanjiang River	Yang et al. 2018
47	*S.wumengshanensis* Li, Mao, Lu & Yan, 2003	*S.tingi* group	Yunnan	Yuanjiang River	Li et al. 2003a
48	*S.xichouensis* Pan, Li, Yang & Chen, 2013	*S.tingi* group	Yunnan	Yuanjiang River	Pan et al. 2013
49	*S.yangzongensis* Chu & Chen, 1977	*S.tingi* group	Yunnan	Nanpanjiang River	Zhao and Zhang 2009
50	*S.yimenensis* Li & Xiao, 2005	*S.tingi* group	Yunnan	Yuanjiang River	Li et al. 2005
51	*S.brevis* Lan & Chen, 1992	*S.cyphotergous* group	Guangxi	Liujiang River	Chen and Lan 1992
52	*S.cyphotergous* (Dai, 1988)	*S.cyphotergous* group	Guizhou	Hongshui River	Huang et al. 2017
53	*S.donglanensis* Zhao, Watanabe & Zhang, 2006	*S.cyphotergous* group	Guangxi	Hongshui River	Zhao et al. 2006
54	*S.donglanensis* Zhou, Liu & Wang, 2011	*S.cyphotergous* group	Guizhou	Liujiang River	Zhou et al. 2011
55	*S.gracilicaudatus* Zhao & Zhang, 2014	*S.cyphotergous* group	Guangxi	Liujiang River	Wang et al. 2014
56	*S.guiyang* Shao, Cheng, Lu, Zhou & Zeng, 2024	*S.cyphotergous* group	Guizhou	Yangtze River	Shao et al. 2024
57	*S.huanjiangensis* Wu, Gan & Li, 2010	*S.cyphotergous* group	Guangxi	Liujiang River	Wu et al. 2010
58	*S.hugeibarbus* Li, Ran & Chen, 2003	*S.cyphotergous* group	Guizhou	Liujiang River	Li et al. 2003b
59	*S.lingyunensis* Li, Xiao & Lu, 2000	*S.cyphotergous* group	Guangxi	Hongshui River	Li et al. 2000
60	*S.longibarbatus* Wang & Chen, 1989	*S.cyphotergous* group	Guizhou; Guangxi	Liujiang River	Wang and Chen 1989
61	*S.luopingensis* Li & Tao, 2002	*S.cyphotergous* group	Yunnan	Nanpanjiang River	Li et al. 2002a
62	*S.macrolepis* Wang & Chen, 1989	*S.cyphotergous* group	Guizhou; Guangxi	Liujiang River	Wang and Chen 1989
63	*S.macrophthalmus* Zhang & Zhao, 2001	*S.cyphotergous* group	Guangxi	Hongshui River	Zhang and Zhao 2001
64	*S.multipunctatus* (Pellegrin, 1931)	*S.cyphotergous* group	Guizhou; Guangxi	Liujiang River; Hongshui River; Wujiang River	Zhao and Zhang 2009
65	*S.punctatus* Lan & Yang, 2017	*S.cyphotergous* group	Guizhou; Guangxi	Liujiang River; Hongshui River	Lan et al. 2017
66	*S.ronganensis* Luo, Huang & Wen, 2016	*S.cyphotergous* group	Guangxi	Liujiang River	Luo et al. 2016
67	*S.sanxiaensis* Jiang, Li, Yang & Chang, 2019	*S.cyphotergous* group	Hubei	Yangtze River	Jiang et al. 2019
68	*S.xunlensis* Lan, Zhan & Zhang, 2004	*S.cyphotergous* group	Guangxi	Liujiang River	Lan et al. 2004
69	*S.yaolanensis* Zhou, Li & Hou, 2009	*S.cyphotergous* group	Guizhou	Liujiang River	Zhou et al. 2009
70	*S.yishanensis* Li & Lan, 1992	*S.cyphotergous* group	Guangxi	Liujiang River	Li and Lan 1992
71	*S.brevifinus* Li, Li & Mayden, 2014	*S.jii* group	Guangxi	Hejiang River	Li et al. 2014
72	*S.guanyangensis* Chen, Peng & Zhang, 2016	*S.jii* group	Guangxi	Guijiang River	Chen et al. 2016
73	*S.guilinensis* Ji, 1985	*S.jii* group	Guangxi	Guijiang River	Zhao and Zhang 2009
74	*S.huangtianensis* Zhu, Zhu & Lan, 2011	*S.jii* group	Guangxi	Hejiang River	Zhu et al. 2011
75	*S.jii* Zhang & Dai, 1992	*S.jii* group	Guangxi	Guijiang River	Zhang and Dai 1992
76	*S.gracilis* Li, 2014	No assignment	Guangxi	Guijiang River	Li and Li 2014
77	*S.luolouensis* Lan, 2013	No assignment	Guangxi	Hongshui River	Lan et al. 2013
78	*S.pingshanensis* Li, Li, Lan & Wu, 2018	No assignment	Guangxi	Liujiang River	Wu et al. 2018
79	*S.wui* Li & An, 2013	No assignment	Yunnan	Mingyihe River	Li and An 2013

During our biodiversity survey in southwestern Guizhou Province, China, in October 2019, a specimen of *Sinocyclocheilus* with normal eyes, scaleless and absence of irregular black markings on the body lateral was collected in a completely dark cave. This specimen was identified to the *S.tingi* species group based on morphological characters. Subsequent morphological examination and molecular evidence suggest that this specimen represents an undescribed species of the *S.tingi* species group within the genus S*inocyclocheilus*. However, between 2019 and 2023, we conducted 16 more surveys in this cave again, none of which revealed any new individuals. Considering the conservation of the species and the rescue of diversity, here we formally describe the species as *Sinocyclocheilusxiejiahuai* sp. nov. based on the single-numbered specimen. Although there is only one specimen of this species, the significance of its discovery is as important as that of the publication of *Sinocyclocheilussanxiaensis* in terms of zoogeography and conservation biogeography (Jiang et al. 2019).

## ﻿Materials and methods

### ﻿Sampling and preservation

The single specimen of the genus *Sinocyclocheilus* were collected in southwestern Guizhou Province, China during a cave fish diversity survey in southern China in October 2019. Gill muscle tissue was preserved in 95% alcohol at -20 °C for molecular analysis. All specimens were fixed in 7% buffered formalin and then transferred to 75% ethanol for long-term storage. Specimen were preserved at Guizhou Normal University, Guiyang City, Guizhou Province, China.

### ﻿Morphological comparison and statistical analysis

The new species can be placed in the *S.tingi* species group based on morphology and can be clearly distinguished from species in the *S.angularis*, *S.cyphotergous*, and *S.microphthalmus*, *S.jii* species groups, e.g., absence of horn-like structures and indistinct elevation at the head-dorsal junction; pectoral fins short, not reaching to pelvic-fin origin; and with serrations along posterior margin of the last unbranched fin of the dorsal fin (Zhao et al. 2009). Therefore, this study focused on morphological comparisons with 26 species within the *S.tingi* species group (Table 2).

**Table 2.** Comparison of the diagnostic features of the new species described here with those selected for the 26 species of the *S.tingi* group and four unassigned species (last four) within the genus Sinocyclocheilus. Grey shading indicates clear difference in character compared to that of *Sinocyclocheilusxiejiahuai* sp. nov.

**Species**	**Body lateral markings**	**Gill rakers**	**Dorsal-fin rays**	**Pectoral-fin rays**	**Anal-fin rays**	**Pelvic-fin rays**	**Caudal -fin rays**	**Lateral-line scales/pores**	**Tip of pectoral fin reaching to ventral fin**	**Tip of pelvic-fin rays reaching to anus**	**Source**
*S.xiejiahuai* sp. nov.	Absent	9	iii, 6½;	i, 13	iii, 5	i, 7	17	74	No	No	This study
*S.aluensis*	Present	5–7	iii, 7	i, 13–16	ii, 5	i, 7–9	15–17	71–75	No	No	Li et al. 2005
*S.angustiporus*	Present	7–9	iv,7	i, 14–16	iii, 5	i, 8	15–16	68–80	NA	No	Zhao and Zhang 2009
*S.anophthalmus*	Absent	7–9	iv,8	i, 15–16	iii, 5	i, 8	16	52–56	Yes	No	Chen et al. 1988a
*S.grahami*	Present	5–8	iii, 7	i, 15–17	iii, 5	i, 8–9	16	61–69	No	No	Zhao and Zhang 2009
*S.guishanensis*	Present	5–6	iii, 7	i, 13–16	iii, 5	i, 7–8	15–16	73–80	No	No	Li et al. 2003a
*S.huaningensis*	Present	6	iii, 7	i, 16	iii, 5	i, 8	16	59–67	No	Yes	Li et al. 1998
*S.huizeensis*	Present	5–6	iii, 7	i, 15–16	iii, 5	i, 10	18	70–73	No	No	Cheng et al. 2015
*S.bannaensis*	Present	5	iii, 8	i, 9	ii, 5	i, 9	16	47	Yes	No	Li et al. 2019
*S.maculatus*	Present	14–17	iii, 7	i, 14–15	iii, 5	i, 7~8	16	81–88	No	No	Zhao and Zhang 2009
*S.maitianheensis*	Present	7–8	iii, 7	i, 14–15	iii, 5	i, 9	18	70–82	No	Yes	Li 1992
*S.malacopterus*	Present	7–9	iii, 7	i, 14–18	iii, 5	i, 9	15–16	67–81	No	No	Chu and Cui 1985
*S.longifinus*	Absent	NA	iii, 7	i, 16	ii, 5	i, 8	17	70–72	Yes	Yes	Li et al. 1998
*S.longshanensis*	Present	15–18	iii, 7	i, 14–15	ii, 5	i, 7–8	16	59–62	No	No	Li et al. 2018
*S.macrocephalus*	Absent	12	iv, 7	i, 15–17	iii, 5	i, 8	16	72–78	No	No	Li 1985
*S.lateristriatus*	Present	7–10	iv, 7	i, 15–16	iii, 5	i, 8	17	75–91	No	No	Li 1992
*S.purpureus*	Present	7–8	iv, 6–7	i, 16	iii, 5	i, 8	NA	63–67	No	No	Li 1985
*S.qiubeiensis*	Present	6–7	iii, 7	i, 14–17	iii, 5	i, 8–9	16	67–81	No	No	Li et al. 2002b
*S.qujingensis*	Absent	6–8	iii, 7	i, 16	iii, 5	i, 8	16	70–79	No	No	Li et al. 2002c
*S.robustus*	Present	9	iv, 7	i, 13–16	iii, 5	i, 7–8	16	72	No	No	Chen et al. 1988b
*S.wumengshanensis*	Present	5–6	iii, 7	16	ii, 5	i, 8	16	67–76	Yes	Yes	Li et al. 2003a
*S.xichouensis*	Present	6	iii, 6–7	i, 14–16	iii, 5	i, 8–9	NA	74–88	Yes	No	Pan et al. 2013
*S.tingi*	Present	7–9	iv, 7	i, 14–16	iii, 5	i, 6–8	16	62–73	No	No	Zhao and Zhang 2009
*S.yangzongensis*	Absent	8–11	iii, 7	i, 16	iii, 5	i, 9	16	71–81	No	No	Zhao and Zhang 2009
*S.yimenensis*	Present	5–7	iii, 7	i, 14–15	ii, 5	i, 8	16-17	70–79	No	No	Li et al. 2005
*S.oxycephalus*	Present	6–7	iv, 7	i, 16	iii, 5	i, 8	17	74–78	No	No	Li 1985
*S.wenshanensis*	Present	7–9	iii, 7	i, 13–15	ii, 5	i, 7–8	14–15	67–72	No	Yes	Yang et al. 2018
*S.macroscalus*	Present	9-10	iv, 7	i, 15–16	iii, 5	i, 8	NA	70-79	No	No	Li 1992
*S.gracilis*	Absent	12	NA	NA	NA	NA	NA	NA	No	No	Li and Li 2014
*S.pingshanensis*	Absent	10–12	iii, 7	i, 13–15	ii, 5	i, 7–8	16	75–78	Yes	No	Wu et al. 2018
*S.luolouensis*	Present	10	iii, 7	i, 13–14	iii, 5	i, 7–8	16–17	40–49	Yes	Yes	Lan et al. 2013
*S.wui*	Absent	7	iii, 7	i, 14–15	ii, 5	i, 7–8	14–15	79–81	No	No	Li and An 2013

We measured 32 morphometric data points (Suppl. material 1) from a total of nine specimens of three species, referenced from Xu et al. (2023). Principal component analysis (PCA) of corrected morphometric measurements and two-dimensional scatter plots were used to explore the relative contribution of specific variables to morphological variation. Prior to PCA analysis, all included measurements were normalized using ratios to standard length (standard length is the ratio to full length) followed by log transformation (Shao et al. 2024). PCA analyses were performed in SPSS 21.0 (SPSS, Inc., Chicago, IL, USA).

### ﻿DNA extraction and sequencing

We sequenced the mitochondrial genomes of 13 species of the genus *Sinocyclocheilus*. Total genomic DNA was extracted from each sample of 95% ethanol-preserved tissue using the Cetyltrimethylammonium Bromide method. For mitogenome sequencing, genomic DNA was fragmented to an appropriate size of 150–500 bp using a Covaris Ultrasonicator. A 400 bp DNA library was constructed based on the Whole Genome Shotgun and the library size. Sequencing was performed on an Illumina NovaSeq 6000 platform using a paired-end 150 bp protocol, generating an average of ~ 5.4 Gb of raw data. The raw data were cleaned using SOAPnuke v. 1.3 (Chen et al. 2018) based on the following criteria: removal of reads with more than 5% N-base content, reads with 50% low-quality bases, and reads with adapter contamination. The process yielded ~ 5.3 Gb of clean data. Mitogenome assembly was performed on the clean data using SPAdes v. 3.13 (parameter: -k 127) (Bankevich et al. 2012), and the assembled contigs were used for BLASTN analysis (BLAST 2.2.30+, parameter: e^-5^) using a reference mitogenome (*Sinocyclocheilusrhinocerous*: KR069119) to identify possible assembly errors. Assembled mitogenomes were annotated for genes using MITOS (Bernt et al. 2013). For mitochondrial cytochrome b (Cyt *b*) and NADH dehydrogenase subunit 4 (ND4) genes, PCR amplifications and sequencing followed Xu et al. (2023).

### ﻿Phylogenetic reconstruction and divergence time estimate

In total, we collected 43 mitochondrial genomes and 76 mitochondrial gene fragments (36 Cyt*b*, 34 ND4, five 16S, three ND5, and three CO1) for phylogenetic reconstruction and estimation of divergence times. Following Wen et al. (2022), we selected *Carassiusauratus*, *Cyprinuscarpio*, *Schizothoraxyunnanensis*, *Onychostomasimum*, *Barbusbarbus*, *Puntiusticto*, *Neolissochilushexagonolepis*, *Garraorientalis*, *Myxocyprinusasiaticus*, and *Daniorerio* as outgroup species (Table 3). All sequences were assembled and aligned using the MUSCLE (Edgar 2004) module in MEGA v. 7.0 (Kumar et al. 2016) with default settings. The best-fit model was obtained based on the Bayesian information criterion computed with PartitionFinder v. 2.1.1 (Lanfear et al. 2017) (Suppl. material 2).

**Table 3.** Localities, voucher information, and GenBank numbers for all samples used.

ID	Species	Location (* type localities)	Voucher number	Mitogenome	Cyt *b*	ND4/16S/ND5/CO1
1	*S.altishoulderus*	Mashan County, Guangxi	–	NC_013186		
2	*S.anatirostris*	–	GZNU20210531002	NC_069226		
3	*S.angularis*	Panzhou City, Guizhou*	GZNU20180420001	MZ636514		
4	*S.angularis*	Baotian Town, Panzhou City, Guizhou	GZNU20180420001	PQ157935		
5	*S.angustiporus*	Xingren County, Guizhou	GZNU20190504001	MZ636515		
6	*S.anophthalmus*	–	–	NC_023472		
7	*S.anshuiensis*	Lingyun County, Guangxi	–	KR069120		
8	*S.aquihornes*	Shuanglongjian town, Qiubei County, Yunnan*	S28	–	**PQ155086**	**PQ155094**
9	*S.bicornutus*	XingrenCounty, Guizhou	–	KX528071		
10	*S.brevibarbatus*	–	GX0064–L20–13	–	MT373106	MW548423
11	*S.brevifinus*	–	–	–	OQ718395	
12	*S.brevis*	–	GX0155	–	MT373105	MW548424
13	*S.cyphotergous*	Luodian County, Guizhou*	GZNU20150819010	OQ319607		
14	*S.convexiforeheadus*	Wenliu Township, Qiubei County, Yunnan*	S30	–	**PQ155090**	**PQ155091**
15	*S.donglanensis*	Donglan County, Guangxi	CA139		AB196440	MW548425
16	*S.furcodorsalis*	Tian’e County, Guangxi	–	GU589570		
17	*S.gracilicaudatus*	–	–	–	OQ718398	
18	*S.grahami*	Kunming City, Yunnan	–	GQ148557		
19	*S.guanyangensis*	–	GX0173	–	MT373108	MW548426
20	*S.guilinensis*	–	GX0073–L17–2	–	MT373104	MW548427
21	*S.guishanensis*	Guishan, Shilin County, Yunnan	XH5401	–	AY854722	AY854779
22	*S.huangtianensis*	–	GX0175	–	MT373109	MW548428
23	*S.huaningensis*	Huaning County, Yunnan	XH3701	–	AY854718	AY854775
24	*S.huanjiangensis*	–	GX0124		MT373103	MW548429
25	*S.hugeibarbus*	Libo County, Guizhou*	GZNU20150120005	MW014319		
26	*S.huizeensis*	Huize County, Yunnan	hrfri2018046	MH982229		
27	*S.hyalinus*	Alugudong, Luxi County, Yunnan	XH4701	–	AY854721	AY854778
28	*S.jii*	Gongcheng County, Guangxi	YNUSJ201308060038	MF100765		
29	*S.jiuxuensis*	Jiuxu Town, Hechi City, Guangxi	XH8501	–	AY854736	AY854793
30	*S.lateristriatus*	Maojiachong, Zhanyi County, Yunnan	XH1102	–	AY854703	AY854760
31	*S.lingyunensis*	–	–	MW411665		
32	*S.longibarbatus*	Libo County, Guizhou*	GZNU2019102022	NC_056194		
33	*S.longihornes*	Hongguo Town, Panzhou City, Guizhou*	GZNU20210503016	–	MZ634123	MZ634125
34	*S.longshanensis*	Shupi Township, Qiubei County, Yunnan*	S22	–	**PQ155085**	**PQ155093**
35	*S.macrocephalus*	Heilongtan, Shilin County, Yunnan	XH0103		AY854683	AY854740 DQ845925
36	*S.macrolepis*	Nandan County, Guangxi	XH8201		AY854729	AY854786
37	*S.macrophthalmus*	Xiaao, Duan County, Guangxi	XH8401		AY854733	AY854790 HM536754 HM536835 HM536889
38	*S.maculatus*	Weiwei Township, Yanshan County, Yunnan	8	–	EU366193	EU366183
39	*S.malacopterus*	Wulong Township, Shizong County, Yunnan*	S43	–	**PQ155088**	**PQ155095**
40	*S.maitianheensis*	Jiuxiang,Yiliang County, Yunnan	XH2301		AY854710	AY854767
41	*S.mashanensis*	–	GX0026–L18–12		MT373107	MW548430
42	*S.microphthalmus*	Lingyun County, Guangxi	NNNU201712001	MN145877		
43	*S.multipunctatus*	Huishui County, Guizhou	–	MG026730		
44	*S.oxycephalus*	Shilin County, Yunnan	YNUSO20160610002	MG686610		
45	*S.purpureus*	Luoping County, Yunnan	IHB:2006638	MW548264		
46	*S.punctatus*	–	–	MW014318		
47	*S.purpureus*	Zhonghe Ying Township, Kaiyuan, Yunnan*	S20	–	**PQ155083**	**PQ155097**
48	*S.qiubeiensis*	Songming, Yunnan	IHB:2006624	NC_063104		
49	*S.qiubeiensis*	Qiubei County, Yunnan*	S21	–	**PQ155084**	**PQ155098**
50	*S.qujingensis*	Huize County, Yunnan	hrfri2018044	MH937706		
51	*S.rhinocerous*	Luoping County, Yunnan	–	KR069119		
52	*S.ronganensis*	Rong’an County, Guangxi	–	KX778473		
53	*S.sanxiaensis*	Guojiaba Town, Zigui County, Hubei*	KNHM 2019000001	OP745534		
54	*S.simengensis*	–	–		OQ718406	
55	*S.tingi*	Fuxian Lake, Yunnan	YNUST201406180002	MG323567		
56	*S.wenshanensis*	Xigu Town, Wenshan, Yunnan	YNUSW20160703016	MW553076		
57	*S.wenshanensis*	Dehou Town, Wenshan City, Yunnan*	S45	–	**PQ155089**	**PQ155100**
58	*S.wumengshanensis*	Xuanwei County, Yunnan	YNUSM20160817008	MG021442		
59	*S.xunlensis*	Huanjiang, Guangxi	IHB:04050268		EU366187	EU366184 HM536752 HM536833 HM536887
60	*S.xiejiahuai* sp. nov.	Hongguo Town, Panzhou City, Guizhou*	S46	**PQ165088**		
61	*S.xingyiensis*	XingyiCity, Guizhou, China*	–		ON573218	
62	*S.xichouensis*	Xingjie Town, Xichou County, Yunnan*	S37	–	**PQ155087**	**PQ155099**
63	*S.yangzongensis*	Yangzonghai Lake, Yunnan	XH6101		AY854725	AY854782 DQ845926
64	*S.yimenensis*	Yimen, Yunnan	IHB:2006646		EU366191	EU366180
65	*S.yishanensis*	Liujiang County, Guangxi	–	MK387704		
66	*S.zhenfengensis*	Zhenfeng County, Guizhou*	GZNU20150112021	MW014317		
67	*S.zhenfengensis*	Zhenfeng County, Guizhou*	S17	–	**PQ155082**	**PQ155096**
68	*S.tianlinensis*	Longping Township, Tianlin County, Guangxi*	S10	–	**PQ155081**	**PQ155092**
69	*S.tianlinensis*	Longping Township, Tianlin County, Guangxi*	GZNU20210531003	**PQ214929**		
**Outgroup**						
70	*Carassiusauratus*	–	–	AB111951		
71	*Cyprinuscarpio*	–	–	JN105357		
72	*Schizothoraxyunnanensis*	–	–	KR780749		
73	*Onychostomasimum*	–	–	KF021233		
74	*Barbusbarbus*	–	–	AB238965		
75	*Puntiusticto*	–	–	AB238969		
76	*Neolissochilushexagonolepis*	–	–	KU380329		
77	*Garraorientalis*	–	–	JX290078		
78	*Myxocyprinusasiaticus*	–	–	AY526869		
79	*Daniorerio*	–	–	KM244705		

Phylogenetic reconstruction and divergence time estimation were performed in BEAST v. 2.4.7 (Bouckaert et al. 2014). In the absence of a reference fossil calibration, we refer to Wen et al. (2022) and Yang et al. (2016): (1) *Sinocyclocheilus* originated at 43.96 million years (Ma) (sigma = 2.8); (2) the most recent common ancestor of *Sinocyclocheilus* occurred at 32.13 Ma (sigma = 2.8); (3) the divergence of the *S.angularis* species group and the *S.tingi* + *S.cyphotergous* species groups occurred at ~ 26.3 Ma (sigma = 4.2). BEAST analyses were run for 40 million generations under an uncorrelated relaxed clock model and a Yule tree prior, sampled every 5000 generations. All calibrations were performed using a normal prior and monophyly. Convergence of the run parameters was checked using Tracer v. 1.7.1 (Rambaut et al. 2018) to ensure that the effective sample size of all parameters was greater than 200. A maximum clade credibility tree was generated using Treeannotator v. 2.4.1 (Bouckaert et al. 2014) by applying a burn-in of 25%. Uncorrected *p*-distances (1000 replicates) based on Cyt *b* gene were calculated in MEGA v. 7.0 (Kumar et al. 2016).

## ﻿Results

### ﻿Phylogenetic analyses, genetic divergence, and divergence time

The length of the aligned sequence was 15671 base pairs (bp), including 16S (1718 bp), 12S (954 bp), tRNAs (1587 bp), ATP6 (684 bp), ATP8 (165 bp), COI (1551 bp), COII (691 bp), COIII (786 bp), Cyt *b* (1142 bp), ND1 (975 bp), BD2 (1045 bp), ND3 (349 bp), ND4 (1381 bp), ND4L (297 bp), ND5 (1824 bp) and ND6 (522 bp). Information on the evolutionary models used for phylogenetic reconstruction is shown in Suppl. material 2.

**Figure 2.**
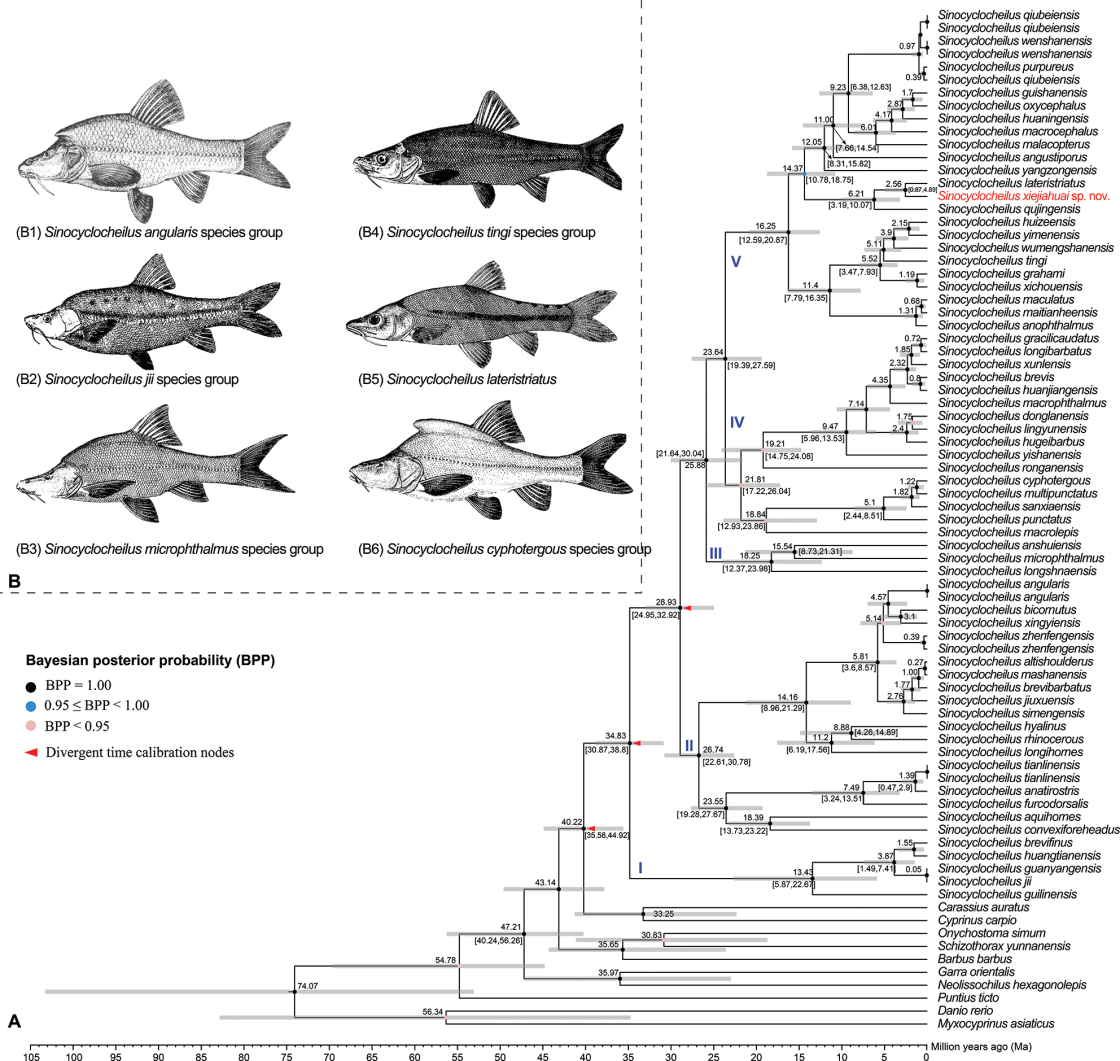
**A** time tree based on mitochondrial genes assessment **B** type species for five species groups. Species photos B1, B3, B4, and B6 from Shan et al. (2000), B2 from Zhang and Dai (1992), and B5 from Li (1992).

The phylogenetic tree reconstructed using BEAST shows that the living *Sinocyclocheilus* can be divided into five major clades, Clades I–V, and is highly resolved (BPP = 1.00) (Fig. 2A). The phylogenetic relationship between the four clades is (Clade I+(Clade II+(Clade III + (Clade IV+ Clade V)))) (Fig. 2A). New species clustered in Clade V, close to *S.lateristriatus*, had a genetic distance of 1.9% at the level of the mitochondrial Cyt *b* (Suppl. material 3).

Divergence time analyses indicate that *Sinocyclocheilus* originated 40.22 Ma (95% highest probability density (HPD): 35.58–44.92 Ma), with its most recent common ancestor occurring at 34.83 (95%HPD: 30.87–38.8 Ma). Divergence of the remaining four clades (Clades II–IV) occurred in the Oligocene to Early Miocene, ~ 23.64–28.93 Ma (95% HPD: 19.39–32.92 Ma). The divergence of the new species from its close relatives occurred at the Pliocene/Pleistocene boundary at ~ 2.56 Ma (95% HPD: 0.87–4.89 Ma), which is older than the divergence of the other sister species (Fig. 2A).

### ﻿Morphological analyses

**Figure 3.**
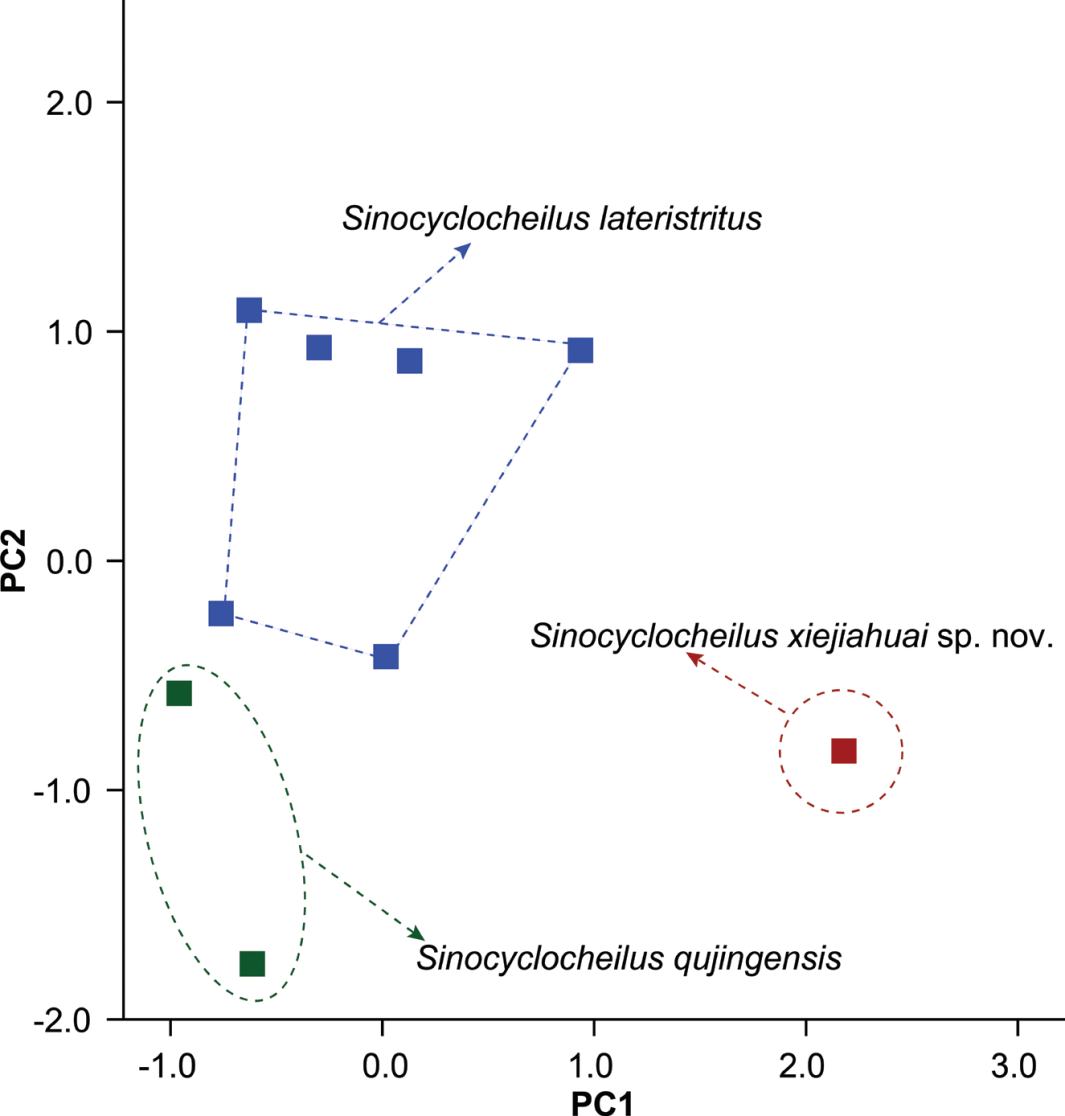
Plot of principal component analysis, scores of *Sinocyclocheilusxiejiahuai* sp. nov., *S.lateristritus*, and *S.qujingensis* based on morphometric data.

A total of five principal component factors with eigenvalues greater than two were extracted based on principal component analysis of the morphometric data (Suppl. material 3). These together accounted for 94.48% of the total variance, with the first principal component (PC1) and second principal component (PC2) accounting for 32.98% and 25.84% of the total variance. In the scatter plot of PC1 versus PC2, the new species *Sinocyclocheilusxiejiahuai* sp. nov. was distinguishable from *S.lateristritus* and *S.qujingensis* on the PC1 axis (Fig. 3). Major morphometric characters loaded on the PC1 axis included body depth, anal-fin length, prepectoral length, pectoral-fin base length, caudal peduncle length, caudal peduncle depth, head width, snout length, eye diameter, interorbital width, distance between anterior nostrils, mouth width, rostral barbel length, and maxillary barbel length (Table 4).

**Table 4.** PCA loadings of five principal components extracted from 34 morphometric data of *S.xiejiahuai* sp. nov. and its related species.

	PC1	PC2	PC3	PC4	PC5
Standard length	0.389	0.508	-0.188	-0.616	0.164
Body depth	0.645	0.419	-0.614	-0.089	0.031
Predorsal length	0.351	0.531	-0.586	0.406	-0.159
Dorsal-fin base length	-0.476	0.765	-0.010	-0.267	0.106
Dorsal-fin length	-0.303	0.877	-0.217	-0.020	0.004
Pre-anal length	-0.383	0.026	-0.791	0.005	0.336
Anal-fin base length	-0.473	0.259	-0.066	0.155	0.648
Anal-fin length	-0.640	0.612	0.265	0.221	0.034
Prepectoral length	-0.820	0.378	-0.159	-0.275	-0.134
Pectoral-fin base length	0.683	0.056	0.499	-0.489	-0.080
Pectoral-fin length	-0.031	0.280	0.695	-0.075	-0.505
Prepelvic length	-0.544	0.741	0.325	0.096	0.030
Pelvic-fin base length	0.588	-0.293	-0.060	0.043	-0.662
Pelvic-fin length	-0.243	0.880	0.029	0.175	-0.363
Caudal peduncle length	0.602	-0.479	0.583	0.112	0.211
Caudal peduncle depth	0.725	0.363	-0.429	-0.356	-0.103
Head length	0.261	0.774	0.511	-0.039	0.180
Head depth	0.571	0.583	-0.409	-0.291	-0.219
Head width	0.727	0.353	-0.257	0.512	-0.004
Snout length	0.625	0.386	0.457	0.259	0.197
Eye diameter	0.643	0.702	0.060	-0.233	0.041
Interorbital width	0.755	0.532	0.057	-0.001	0.191
Prenostril length	0.852	0.336	0.008	0.063	0.348
Distance between posterior nostrils	0.561	-0.141	0.460	-0.393	0.278
Upper jaw length	-0.535	0.436	0.524	0.176	0.084
Lower jaw length	-0.562	0.649	0.151	0.248	0.072
Mouth width	0.731	0.415	-0.384	0.202	0.022
Rostral barbel length	0.639	0.369	0.403	0.477	-0.010
Maxillary barbel length	0.634	-0.022	0.719	0.192	-0.146
Distance from the pectoral-fin origin to the pelvic-fin origin	0.459	-0.352	-0.581	0.445	-0.073
Distance from the pelvic-fin origin to the anal-fin origin	0.456	-0.582	0.054	0.046	0.460
Eigenvalues	6.887	10.361	1.049	0.689	0.979
Percentage of total variance	32.981	25.837	17.032	7.820	6.814
Cumulative percentage	32.981	58.817	75.850	83.669	90.483

### ﻿Morphological comparison

Based on morphology and phylogeny, the new species *Sinocyclocheilusxiejiahuai* sp. nov. was assigned to the *S.tingi* group, and a detailed morphological comparison is shown in Table 2.

*Sinocyclocheilusxiejiahuai* sp. nov. can be distinguished from the 24 species belonging to the *S.angularis* and *S.microphthalmus* groups by the absence of horn-like structures and indistinct elevation at the head-dorsal junction, pectoral fins tip not reaching to pelvic-fin origin (vs presence of horn-like structures and pectoral fins long and not reaching to pelvic-fin origin); from the five species belonging to the *S.jii* species group by with serrations along posterior margin of the last unbranched fin of the dorsal fin (vs absent) (Zhao et al. 2009), and from the 21 species belonging to the *S.cyphotergous* species group by pectoral fins tip not reaching to pelvic-fin origin (vs usually reaching to pelvic-fin origin).

For the 26 species of the *S.tingi* group, new species can be distinguished by a series of morphological characters. By lacking irregular markings on the body lateral, the new species can be distinguished from *S.aluensis*, *S.angustiporus*, *S.bannaensis*, *S.grahami*, *S.guishanensis*, *S.huaningensis*, *S.huizeensis*, *S.lateristriatus*, *S.longshanensis*, *S.macrocephalus*, *S.maculatus*, *S.maitianheensis*, *S.malacopterus*, *S.oxycephalus*, *S.purpureus*, *S.robustus*, *S.wumengshanensis*, *S.xichouensis*, *S.tingi*, *S.yimenensis*, and *S.wenshanensis*. New species differs from *S.anophthalmus* by eyes present (vs absent) and lateral line pores 74 (vs 52–56); from *S.longifinus*, *S.qujingensis*, and *S.yangzongensis* by six branched dorsal-fin rays (vs 7) and seven branched pelvic-fin rays (vs 8 or 9). The new species can be further distinguished from *S.qujingensis* and *S.yangzongensis* by 13 branched pectoral-fin rays (vs 16), and from *S.longifinus* by the tip of the pectoral fin not reaching to pelvic-fin origin (vs reaching to pelvic-fin origin).

For the four species not placed in any species group, new species differed from *S.luolouensis* by eye normal (vs eyes reduced) and pectoral fins tip not reaching to pelvic-fin origin (vs .beyond pelvic-fin origin) (Lan et al. 2013), from *S.gracilis* by having nine rakers on the first gill arch (vs 12) and body depth of 13% of standard length (vs 21.0–23.8%), from *S.pingshanensis* and *S.gracilis* by with serrations along posterior margin of the last unbranched fin of the dorsal fin (vs absent) and nine rakers on the first gill arch (vs 10–12), and from *S.wui* by three unbranched anal-fin rays (vs 2), 13 branched pectoral-fin rays (vs 14–15), and 17 branched caudal-fin rays (vs 14–15).

### ﻿Taxonomic account

#### ﻿ Sinocyclocheilus xiejiahuai

Taxon classificationAnimaliaCypriniformesCyprinidae

Luo, Fan, Xiao & Zhou sp. nov.

880A5938-ED0B-509E-A0DC-89F279C85B40

https://zoobank.org/B3636299-814D-4EE8-92F4-CA48078A7581

Fig. 4 , Table 5

##### Material examined.

***Holotype*.** GZNU20230304001, total length 242.8 mm (TL), standard length 201.8 mm (SL), • Hongguo Town, Panzhou City, Guizhou Province, China; 25.6576°N, 104.4044°E; ca 1852 m a.s.l.; collected on October 2, 2021.

##### Diagnosis.

*Sinocyclocheilusxiejiahuai* sp. nov. can be distinguished from all other congeners by the following combination of characters: (1) absence of horn-like structures and indistinct elevation at the head-dorsal junction; (2) absence of irregular black markings on the body lateral and scaleless; (3) eyes large, eye diameter 13% of head length; (4) dorsal-fin rays, iii, 6½, last unbranched ray strong, with serrations along posterior margin; (5) pectoral-fin rays, i, 13; (6) anal-fin rays, iii, 5; (7) pelvic-fin rays, i, 7; (8) lateral line pores 74; (9) gill rakers well developed, nine on first gill arch; (10) pectoral fins short, tip not reaching to pelvic-fin origin (Table 5).

**Table 5.** Morphological characteristics and measurements of *Sinocyclocheilusxiejiahuai* sp. nov., *S.qujingensis*, and *S.lateristritus*.

	*S.xiejiahuai* sp. nov. (*n* = 1)	*lateristritus* (*n* = 2)		*S.qujingensis* (*n* = 6)	
	Range	Range	Mean ± SD	Range	Mean ± SD
Total length	240.0	73.2–87.5	80.8 ± 5.2	53.3–140.1	96.7 ± 61.4
Standard length	201.0	59.3–71.0	64.9 ± 4.1	43.3–98.1	70.7 ± 38.7
Body depth	56.7	13.0–18.4	15.3 ± 1.9	9.9–19.2	14.6 ± 6.6
Predorsal length	108.6	30.9–39.4	34.1 ± 3.0	22.3–50.0	36.1 ± 19.6
Dorsal-fin base length	24.7	8.5–9.6	9.2 ± 0.4	5.9–12.3	9.1 ± 4.5
Dorsal-fin length	40.0	12.7–16.7	15.2 ± 1.5	10.2–19.4	14.8 ± 6.4
Pre-anal length	140.4	42.0–49.2	45.5 ± 2.4	30.9–68.9	49.9 ± 26.9
Anal-fin base length	17.4	5.2–6.8	5.8 ± 0.5	4.0–8.4	6.2 ± 3.1
Anal-fin length	28.4	9.8–13.3	11.6 ± 1.2	7.3–16.3	11.8 ± 6.4
Prepectoral length	53.0	17.1–19.7	18.4 ± 0.9	12.7–27.2	19.9 ± 10.3
Pectoral-fin base length	8.9	2.1–3.1	2.5 ± 0.5	1.4–3.0	2.2 ± 1.2
Pectoral-fin length	32.9	8.9–16.0	13.0 ± 2.9	7.2–17.5	12.3 ± 7.3
Prepelvic length	98.1	29.9–36.0	33.3 ± 2.1	21.8–49.1	35.5 ± 19.3
Pelvic-fin base length	8.9	2.0–3.3	2.5 ± 0.5	1.8–4.2	3.0 ± 1.7
Pelvic-fin length	25.5	8.9–13.5	11.0 ± 1.6	6.5–13.7	10.1 ± 5.1
Caudal peduncle length	49.5	12.3–16.6	14.0 ± 1.6	8.4–22.6	15.5 ± 10.1
Caudal peduncle depth	24.7	6.4–8.5	7.1 ± 0.7	4.6–9.2	6.9 ± 3.2
Head length	57.1	16.4–21.0	19.2 ± 1.6	11.8–26.3	19.0 ± 10.3
Head depth	40.3	11.3–15.2	12.5 ± 1.4	8.1–16.1	12.1 ± 5.6
Head width	33.1	7.8–11.2	9.3 ± 1.2	5.6–13.6	9.6 ± 5.7
Snout length	21.0	4.7–6.8	6.3 ± 0.8	3.3–8.7	6.0 ± 3.8
Eye diameter	6.7	1.8–2.4	2.1 ± 0.2	1.2–2.4	1.8 ± 0.9
Interorbital width	18.6	4.4–6.4	5.5 ± 0.7	3.4–7.1	5.2 ± 2.6
Prenostril length	13.1	2.3–3.5	3.0 ± 0.4	1.7–3.5	2.6 ± 1.3
Distance between posterior nostrils	12.9	2.9–4.8	3.6 ± 0.7	2.4–4.8	3.6 ± 1.7
Upper jaw length	13.7	4.8–5.7	5.3 ± 0.4	3.2–7.6	5.4 ± 3.1
Lower jaw length	12.1	4.4–5.4	4.9 ± 0.4	3.0–6.8	4.9 ± 2.7
Mouth width	17.2	4.4–6.1	5.0 ± 0.7	3.1–6.8	5.0 ± 2.6
Rostral barbel length	24.6	4.8–9.8	7.1 ± 1.6	2.2–9.5	5.9 ± 5.2
Maxillary barbel length	30.2	5.3–12.0	8.0 ± 2.8	2.5–11.8	7.1 ± 6.6
Distance from the pectoral-fin origin to the pelvic-fin origin	42.2	11.4–14.2	12.7 ± 0.9	8.7–20.1	14.4 ± 8.0
Distance from the pelvic-fin origin to the anal-fin origin	38.4	9.5–10.8	9.9 ± 0.5	6.2–16.4	11.3 ± 7.2

##### Description.

Body fusiform, moderately elongate and compressed. Dorsal profile convex from nape to dorsal-fin, greatest body depth at dorsal-fin insertion, ventral profile slightly concave, tapering gradually toward the caudal-fin, greatest body depth slightly anterior to dorsal-fin insertion.

Head short, compressed laterally, length longer than maximum head width, depth longer than maximum head width. Eyes present, eye diameter 13% of head length (HL), interorbital distance larger than distance between posterior nostrils. Snout short, U-shaped, and projecting beyond lower jaw in dorsal view, length 37% of HL. Mouth subterminal, with slightly projecting upper jaw. Two pairs of nostrils, anterior and posterior nostrils close set, nares at 2/3 between snout tip and anterior margin of eye, anterior nares possessing an anterior rim with a posterior fleshy flap forming a half-tube. Two pairs of barbels, rostral barbels short, not reaching the anterior edge of operculum when extended backwards, maxillary barbel slightly long compared to rostral barbel, beyond the anterior edge of operculum when extended backwards (Table 5).

**Figure 4.**
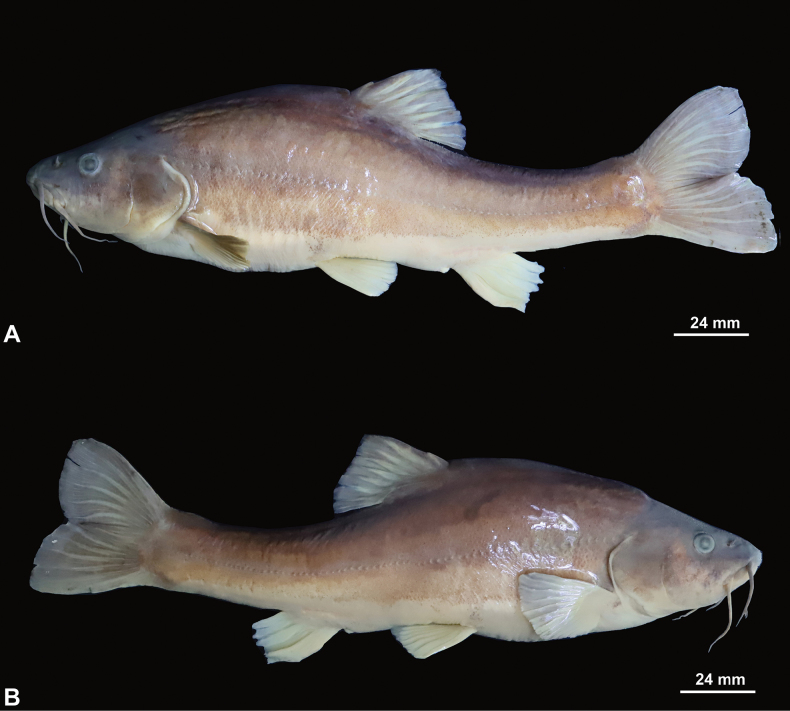
Lateral view of adult holotype GZNU20230304001 of *Sinocyclocheilusxiejiahuai* sp. nov. in preservative **A** left side **B** right side.

Dorsal fin rays iii, 6½, pectoral fin rays i,13, pelvic fin rays i, 7, anal fin rays iii, 5, and 13 branched caudal fin rays. Dorsal fin long, 24% of SL, less than head length, distal margin truncated, origin posterior to pelvic fin insertion, situated slightly anterior to midpoint between snout tip and the caudal fin base, last unbranched ray strong, softening toward tip, with serrations along posterior margin, first branched ray longest, shorter than HL, tip reaching to the vertical of the anus. Pectoral fin developed, distal margin rounded, length slightly small than HL, 16% of SL, tips beyond 2/3 of the distance between pectoral-fin origin and pelvic-fin origin, tips not reaching to pelvic fin-origin. Pelvic fin moderately developed, distal margin rounded, length 14% of SL, and tips not reaching to anus. Anal fin short, 15% of SL, distal margin truncated, origin close to the anus, tips not reaching to caudal fin base. Caudal peduncle well developed, length 52.4 mm, depth 23.4 mm, and without adipose crests along both dorsal and ventral sides. Caudal fin slight forked, upper lobe equal in length to the lower one, tips rounded.

Body non-scale, lateral line pores 74. Complete lateral line, slightly curved, curved downward at the anus position, originating from posterior margin of operculum and extending to end of caudal peduncle.

##### Coloration.

In 7% formalin solution, the specimen was grayish brown overall, with each fin pale yellow.

##### Geographical distribution and habitat.

*Sinocyclocheilusxiejiahuai* sp. nov. is the only vertical cave found at an altitude of 2276 m in Hongguo Town, Panzhou City, Guizhou Province, China, some distance away. The discovery site is within the Beipanjiang River Basin (Fig. 1). There is no light in the cave. Individuals were distributed in a small pool ~ 25 m from the cave entrance. The pool is ~ 1.8 m wide and 80 cm deep, and the water temperature at the time of collection was ~ 16 °C and pH 7.4. Inside the cave, the species of *S.xiejiahuai* sp. nov. is symbiotic with *S.longicornus* (*S.angularis* group) and *Triplophysapanzhouensis*. The arable land outside the cave is mainly cultivated with maize, wheat, and potatoes.

##### Etymology.

The specific name *xiejiahuai* is in honor of Professor Jia-Hua Xie (谢家骅), for his contribution to zoological research in China. Before retiring from Guizhou Normal University, he described *S.angustiporus*, the first species distributed in Guizhou within the *S.tingi* species group, and his work has been an important contribution to the study of zoology in Guizhou, especially the conservation of critically endangered species. We propose the common English name “Xie’s Golden-lined Fish” and the Chinese name “Xiè Shì Jīn Xiàn Bā (谢氏金线鲃).”

## ﻿Discussion

Based on previous records, the genus *Sinocyclocheilus* (Fang, 1936) was recorded with 79 species (Zhao and Zhang 2009; Xu et al. 2023; Luo et al. 2023; Shao et al. 2024), all of which are endemic to southwestern China, and the description of the new species in this study increases it to 80 species. Up to now, there are 27 species in the *tingi* group, mainly distributed in eastern Yunnan and western Guizhou. *Sinocyclocheilusxiejiahuai* sp. nov. is the third species of the *S.tingi* species group discovered in Guizhou with *S.angustiporus* and *S.robutus*. Although the description of this new species is based on a single specimen and some measurements may not be sufficient, the fin characteristics (see morphological comparisons above) and genetic differences support the validity of this new species. The new species is phylogenetically close to *S.lateristriatus* and *S.qujingensis* (Fig. 2), with genetic distances of 1.9% and 3.1% (Suppl. material 3), which was greater than that between sister species of the same genus, e.g., 1.2% between *S.yimenensis* and *S.huizeensis* (Suppl. material 2). The new species co-inhabits a cave with *S.longicornus*, and is the first report of the sympatric distribution of the *S.tingi* and *S.angularis* groups. Furthermore, our phylogenetic tree suggests that *S.longshanensis* should be assigned to the *S.microphthalmus* species group.

Our reconstructed divergence times are similar to those of Wen et al. (2022) who discussed the origin and early diversification of the genus *Sinocyclocheilus*. Time-tree-based results suggest that *Sinocyclocheilus* originated in the late Eocene, with its most recent common ancestor/Clade I occurring at 34.83 Ma, and that divergence of the remaining clades was centered in the Oligocene to early Miocene, ca 19.39–32.92 Ma (Fig. 2). The origin and early divergence events are well-coupled with the rapid uplift of the Qinghai-Tibetan Plateau during the Oligocene-middle Miocene (Ding et al. 2022). Middle Miocene Climate Optimum (17–14 Ma), the monsoon climate brings precipitation to promote the development of caves in the karst region (Farnsworth et al. 2019), which increases the ecological opportunities for the formation of cave fishes within the *Sinocyclocheilus*. We also observed that *Sinocyclocheilusxiejiahuai* sp. nov., *S.lateristriatus*, and *S.qujingensis* are distributed in the Nanpanjiang River basin in close phylogenetic and geographic proximity, and this congruence may indicate that the formation of these species, and even of species in the *S.tingi* group, is related to historical drainage changes resulting from the uplift of the Yunnan-Guizhou Plateau since the Late Miocene (Zhang et al. 2020). Thus, geographic isolation from historical drainage changes has shaped the formation of species diversity in the *S.tingi* species group (Mao et al. 2021,^2022^; Zhao and Zhang 2009; Wen et al. 2022).

This new species is presently restricted to its type locality. Given that its habitat borders the urban area of Panzhou, which is experiencing rapid urbanization, there is a significant risk of habitat disturbance and destruction in the near future. In the last five years, we have conducted a total of 16 field surveys at the type locality, and no new individuals have been detected except for the first one, suggesting that the population size of this species is very small. The Chinese government listed all species of *Sinocyclocheilus* endemic to China as second-class of the national protected animals on 5 February 2021 (National Forestry and Grassland Administration & National Park Administration, 2021). Therefore, the new species has strict legal protection in China, and the local government should strengthen publicity about the protection of this species to avoid it being caught and trafficked. In addition to the small population size, the following threats to the habitat of the new species include declining water levels in caves, pesticide use, domestic waste, and as potential land for urban construction. Therefore, we suggest the species may be eligible for listing as Endangered (B1ab (i, ii, iii) + 2ab (i, ii, iii)) in the IUCN Red List of Threatened Species.

## Supplementary Material

XML Treatment for Sinocyclocheilus xiejiahuai

## ﻿Additional information

### Author ORCIDs

Cui Fan https://orcid.org/0009-0002-8039-649X

Man Wang https://orcid.org/0000-0001-6201-1811

Jia-Jia Wang https://orcid.org/0009-0007-8637-5469

Tao Luo https://orcid.org/0000-0003-4186-1192

Jia-Jun Zhou https://orcid.org/0000-0003-1038-1540

Jiang Zhou https://orcid.org/0000-0003-1560-8759

### Data availability

All of the data that support the findings of this study are available in the main text or Supplementary Information.

## Supplementary materials

Supplementary material 1 Morphometric data for nine *S.tingi* group species Data type docx https://binary.pensoft.net/file/1147717This dataset is made available under the Open Database License (http://opendatacommons.org/licenses/odbl/1.0/). The Open Database License (ODbL) is a license agreement intended to allow users to freely share, modify, and use this Dataset while maintaining this same freedom for others, provided that the original source and author(s) are credited.Cui Fan, Man Wang, Tao Luo, Jia-Jun Zhou, Ning Xiao, Jiang Zhou

Supplementary material 2 The best model obtained using PartitionFinder v. 2.1.1 evaluated under the Bayesian information criterion Data type docx https://binary.pensoft.net/file/1147718This dataset is made available under the Open Database License (http://opendatacommons.org/licenses/odbl/1.0/). The Open Database License (ODbL) is a license agreement intended to allow users to freely share, modify, and use this Dataset while maintaining this same freedom for others, provided that the original source and author(s) are credited.Cui Fan, Man Wang, Tao Luo, Jia-Jun Zhou, Ning Xiao, Jiang Zhou

Supplementary material 3 Uncorrected *p*-distance (%) between 69 species of the genus *Sinocyclocheilus* based on mitochondrial genes Data type xlsx https://binary.pensoft.net/file/1147719This dataset is made available under the Open Database License (http://opendatacommons.org/licenses/odbl/1.0/). The Open Database License (ODbL) is a license agreement intended to allow users to freely share, modify, and use this Dataset while maintaining this same freedom for others, provided that the original source and author(s) are credited.Cui Fan, Man Wang, Tao Luo, Jia-Jun Zhou, Ning Xiao, Jiang Zhou
