# Supplementary material for: ﻿Sinocyclocheilusxiejiahuai (Cypriniformes, Cyprinidae), a new cave fish with extremely small population size from western Guizhou, China
Source: Zookeys. 2024 Oct 3;1214:119–41. doi: 10.3897/zookeys.1214.127629 (PMC11467493; doi:10.3897/zookeys.1214.127629)
Supplement: Supplementary material 1 — Morphometric data for nine S.tingi group species [file zookeys-1214-119_article-127629__-s001.docx]

**Table S1**. Raw morphometric data for *Sinocyclocheilus xiejiahuai* sp. nov., *S. qujingensis*, and *S.* *lateristritus*.

|  | *S. xiejiahuai* sp. nov. | *S. qujingensis* | | *S. lateristritus* | | | | | |
| --- | --- | --- | --- | --- | --- | --- | --- | --- | --- |
|  | GZNU20230  304001 | GZNU2023  0825001 | GZNU2023  0825002 | GZNU2023  0825003 | GZNU2023  0825004 | GZNU2023  0825005 | GZNU2023  0825006 | GZNU2023  0825007 | GZNU2023  0825008 |
| Dorsal fin | iii, 6½ | iii, 7 | iii, 7 | iv,7 | iv,7 | iv,7 | iv,7 | iv,7 | iv,7 |
| Pectoral fin | i, 13 | i,16 | i,16 | i,13 | i,13 | i,13 | i,13 | i,13 | i,13 |
| Caudal fin | 17 | 16 | 16 | 16 | 16 | 16 | 17 | 18 | 18 |
| Anal fin | iii, 5 | ii,5 | ii,5 | iii,5 | iii,5 | iii,5 | iii,5 | iii,5 | iii,5 |
| Pelvic fin | i, 7 | i,8 | i,8 | i,8 | i,7 | i,7 | i,8 | i,8 | i,8 |
| Total length | 240.03 | 140.11 | 53.28 | 87.47 | 80.53 | 81.4 | 76.9 | 85.03 | 73.19 |
| Standard length | 201.04 | 98.13 | 43.33 | 71.01 | 63.43 | 65.43 | 62.69 | 67.75 | 59.3 |
| Body depth | 56.69 | 19.24 | 9.93 | 18.39 | 16.38 | 14.59 | 15.48 | 13.03 | 13.78 |
| Predorsal length | 108.56 | 49.96 | 22.29 | 39.35 | 33.81 | 35.58 | 33.27 | 32.03 | 30.85 |
| Dorsal-fin base length | 24.74 | 12.27 | 5.88 | 9.62 | 9.23 | 9.24 | 9.45 | 9.21 | 8.53 |
| Dorsal-fin length | 39.95 | 19.35 | 10.24 | 16.69 | 15.88 | 16.28 | 15.65 | 14.27 | 12.65 |
| Pre-anal length | 140.41 | 68.89 | 30.9 | 49.19 | 45.46 | 45.78 | 43.95 | 46.59 | 41.98 |
| Anal-fin base length | 17.38 | 8.38 | 3.99 | 5.48 | 5.68 | 6.77 | 5.76 | 5.86 | 5.19 |
| Anal-fin length | 28.37 | 16.26 | 7.25 | 11.74 | 10.84 | 13.3 | 12.08 | 11.57 | 9.79 |
| Prepectoral length | 52.95 | 27.21 | 12.65 | 19.7 | 17.81 | 18.65 | 18.53 | 18.84 | 17.09 |
| Pectoral-fin base length | 8.92 | 3.02 | 1.37 | 2.85 | 2.11 | 2.07 | 2.51 | 3.14 | 2.05 |
| Pectoral-fin length | 32.87 | 17.48 | 7.18 | 16.01 | 8.88 | 13.52 | 13.93 | 15.57 | 10.03 |
| Prepelvic length | 98.07 | 49.11 | 21.79 | 35.99 | 32.51 | 34.04 | 32.54 | 34.57 | 29.9 |
| Pelvic-fin base length | 8.86 | 4.2 | 1.75 | 3.32 | 2.33 | 2.23 | 2.51 | 2.63 | 2.01 |
| Pelvic-fin length | 25.49 | 13.66 | 6.51 | 13.47 | 10.42 | 11.97 | 11.34 | 9.78 | 8.86 |
| Caudal peduncle length | 49.52 | 22.61 | 8.35 | 15.05 | 13.46 | 13.62 | 12.85 | 16.64 | 12.32 |
| Caudal peduncle depth | 24.68 | 9.2 | 4.64 | 8.45 | 7.19 | 6.49 | 7.08 | 6.83 | 6.39 |
| Head length | 57.14 | 26.33 | 11.75 | 20.96 | 19.02 | 19.58 | 18.7 | 20.47 | 16.38 |
| Head depth | 40.32 | 16.05 | 8.12 | 15.15 | 12.43 | 11.92 | 12.1 | 11.91 | 11.32 |
| Head width | 33.12 | 13.63 | 5.55 | 11.2 | 9.49 | 9.94 | 8.98 | 8.51 | 7.82 |
| Snout length | 21.02 | 8.69 | 3.26 | 6.83 | 6.36 | 6.42 | 6.43 | 6.79 | 4.72 |
| Eye diameter | 6.71 | 2.37 | 1.15 | 2.44 | 2.1 | 1.96 | 2.08 | 2.07 | 1.75 |
| Interorbital width | 18.62 | 7.06 | 3.37 | 6.4 | 5.77 | 5.6 | 5.11 | 5.72 | 4.38 |
| Prenostril length | 13.05 | 3.54 | 1.68 | 3.52 | 3.21 | 3.3 | 2.73 | 3.09 | 2.34 |
| Distance between posterior nostrils | 12.92 | 4.75 | 2.36 | 3.92 | 3.54 | 3.41 | 3.01 | 4.82 | 2.92 |
| Upper jaw length | 13.74 | 7.62 | 3.21 | 5.69 | 5.13 | 5.72 | 4.79 | 5.72 | 4.94 |
| Lower jaw length | 12.13 | 6.77 | 3.01 | 5.31 | 4.94 | 5.43 | 4.43 | 4.86 | 4.53 |
| Mouth width | 17.17 | 6.8 | 3.1 | 6.11 | 5.33 | 5.01 | 4.48 | 4.65 | 4.37 |
| Rostral barbel length | 24.62 | 9.54 | 2.19 | 9.78 | 7.5 | 7.48 | 6.16 | 7.11 | 4.83 |
| Maxillary barbel length | 30.17 | 11.78 | 2.48 | 11.19 | 5.99 | 7.18 | 6.4 | 11.99 | 5.34 |
| Distance from the pectoral-fin origin to the pelvic-fin origin | 42.15 | 20.07 | 8.73 | 14.16 | 12.51 | 12.96 | 12.29 | 12.73 | 11.39 |
| Distance from the pelvic-fin origin to the anal-fin origin | 38.37 | 16.37 | 6.23 | 9.69 | 9.57 | 9.74 | 9.86 | 10.77 | 9.53 |
