# Supplementary material for: ﻿Sinocyclocheilusxiejiahuai (Cypriniformes, Cyprinidae), a new cave fish with extremely small population size from western Guizhou, China
Source: Zookeys. 2024 Oct 3;1214:119–41. doi: 10.3897/zookeys.1214.127629 (PMC11467493; doi:10.3897/zookeys.1214.127629)
Supplement: Supplementary material 2 — The best model obtained using PartitionFinder v. 2.1.1 evaluated under the Bayesian information criterion [file zookeys-1214-119_article-127629__-s002.docx]

**Table S2** The best model obtained using PartitionFinder 2.1.1 evaluated under the Bayesian information criterion.

| ID | Best fit model | Partitions |
| --- | --- | --- |
| 1 | GTR+I+G | 12S, 16S |
| 2 | GTR+I+G | tRNAs |
| 3 | GTR+I+G | Nd4l, Cyt b, ND4, ND1, ATP6, ND3 |
| 4 | GTR+I+G | ND2, ND5, ATP8 |
| 5 | TVM+I+G | COII, COIII, COI |
| 6 | HKY+I+G | ND6 |
